# Is Singing Under the Christmas Tree Psychologically Recommended? A Scientific Evaluation

**DOI:** 10.32872/cpe.10841

**Published:** 2022-12-22

**Authors:** Philipp Kanske, Winfried Rief

**Affiliations:** 1 Clinical Psychology and Behavioral Neuroscience, Faculty of Psychology, Technische Universität Dresden, Dresden, Germany; 2 Division of Clinical Psychology and Psychotherapy, Department of Psychology, Philipps-University of Marburg, Marburg, Germany

Clinical Psychology in Europe CPE wants to present latest scientific findings, but also highlight their societal impact, and practical relevance. Following the tradition of our first three years, we integrate these aims in a special Christmas editorial, that can be taken seriously, but there is no need to be overly serious with it.

Many European families build a Christmas tree into a living room, although this room was kept clean and proper for the other times of the year, and no dirt from outside was allowed. This surprising activity for inside decoration follows old Egyptian, Chinese, Jewish and Northern tribal traditions to put some green into buildings during cold winter days. However, it is unique that these trees seem to trigger some urgent need to sing along, preferably together in families. We will analyze whether, from a psychological perspective, it can be recommended to follow this urgent need, or whether we should give priority to stop this tradition.

It is not easy to find someone who does not know at least one Christmas carol. Why is that? If anything, it suggests that singing under the Christmas tree is not particularly aversive. In fact, for most people singing is surprisingly fun; using a pre- to post-design to evaluate singing, your mood seems to improve (Schladt et al., 2017). And it is not the same if you just listen to music, singing yourself is what seems to do the trick (Kreutz et al., 2004). So, dig up all those Christmas carols from memory and sing to your heart’s content?

Now there is one further ingredient that may make the festive singing so pleasurable. The positive mood effect is considerably increased by singing together with others (Schladt et al., 2017). This could be due to a whole range of social effects of joint singing. Singing with others seems to have an “ice-breaker effect”. Faster than other group activities like crafting, it will increase social bonding and felt closeness (Pearce et al., 2015), potentially because performing music together, requires a considerable amount of social coordination. In order to really sing together, you need to anticipate the sounds produced by others, divide attention between yourself and others and constantly adjust your timing to that of the group (Keller, 2008). This social attentiveness and adaptation increases group cohesion and accordingly, group singing even promotes feelings of social inclusion (Welch et al., 2014).

Christmas is the feast of charity. According to Christian tradition, Jesus was born in a stable and the big churches take the occasion of Christmas to collect money for people in need. Singing could actually benefit such altruistic behavior. It enhances empathy, the capacity to share others’ suffering, and also compassionate feelings for others (McDonald et al., 2022). These social emotions, in turn, increase people’s willingness to help, especially when the other is in need (Lehmann et al., 2022). Maybe this is a reason why churches of different traditions also encourage to sing along.

You probably learned the songs that you are singing already as a child. And this is part of the reason why Christmas carols may have a particular magic about them. In contrast to music that we encountered later in life, the songs we were exposed to as children have a special potential to calm us in the face of stress and act as emotional regulators (Gabard-Durnam et al., 2018). Already at six months of age, we seem to prefer our mother singing to us compared to her speaking (Nakata & Trehub, 2004). And singing with others leads to spontaneous cooperative and helpful behavior in four-year-olds (Kirschner & Tomasello, 2010). So take some time to sing with your kids. It will not only improve your mood, but also help in creating some peace and harmony in the family. This could be a helpful game changer if other education attempts have failed.

Even on a bodily level, music in general and singing in groups in particular have astonishing effects. It increases secretory immunoglobulin A, a marker of immune competence that can only be helpful at the height of the latest flu wave when winter really hits and in the late outbreaks of the COVID pandemic (Kreutz et al., 2004).

The broad positive effects of singing have led to the development of a number of clinical interventions making use of mainly group singing for diverse health conditions ranging from somatic (e.g. Reagon et al., 2017) to neurodegenerative (Baird, 2018) and mental health conditions (Williams et al., 2018). Among others, depression could be shown to be reduced during an eight weeks group singing intervention (Petchkovsky et al., 2013). Meta-analytically, group singing effects for mental health conditions reach moderate to large effect sizes in wellbeing and mental health improvements, mainly attributable to improved emotional states, sense of belonging and self-confidence in patients (Williams et al., 2018).

There seems to be little to no downside to singing and given that almost everyone knows a Christmas carol, Christmas might really be the one occasion to actually do it, for yourself, your family, your children and their children, since it is the early songs we learn that we will never forget. Therefore, the conclusion of this scientific evaluation is quite straight forward: just do it, let’s sing together.

On behalf of the whole CPE editorial board, we wish you a relaxing time of the year, and a happy and peaceful new year 2023.
